# Indolent keratitis due to fungus of Malbranchea species. A case report

**DOI:** 10.1016/j.amsu.2020.11.065

**Published:** 2020-11-27

**Authors:** Ashjan Yousef Bamahfouz, Abdulrahman Ali Alsaidi, Ibrahim Jameel Alharbi, Eman Abdulraheem Elsebaei, Ayat Mohammed Aldosari, Ahmed Gamil Farahat, Renad Turki Alhazmi

**Affiliations:** a Department of Ophthalmology, College of Medicine, Umm Al-Qura University, Makkah, Saudi Arabia; b Department of Ophthalmology, Security Forces Hospital, Makkah, Saudi Arabia; c Department of Ophthalmology, Ministry of National Guard Health Affairs, King Abdul-Aziz Medical City, Jeddah, Saudi Arabia; d Department of Ophthalmology, King Abdullah Medical City, Makkah, Saudi Arabia

**Keywords:** Case report, Fungal keratitis, *Malbranchea* species, Corneal ulcer, Diabetes

## Abstract

**Introduction:**

Keratitis caused by saprophytic fungi is on the rise in rural areas, often caused by ocular trauma with wooden objects. Early detection of causative organisms and sustained, supervised management can prevent visual disabilities.

**Case presentation:**

A middle-aged patient from a rural, semi-arid region who presented with pain, redness, and a foreign-body sensation in his left eye resulting from a corneal ulcer induced by trauma from a wooden stick. Due to a history of uncontrolled diabetes and progression of his corneal lesions, he was admitted to our institution for treatment of infectious keratitis. Microbiological examination of corneal scrapings revealed thin, septate hyaline hyphae without conidia or conidiophores, and the patient was diagnosed with a fungal keratitis caused by a *Malbranchea* species. Though the patient initially responded to treatment with topical natamycin, his condition worsened. He was subsequently successfully treated with topical amphotericin B (1 mg/mL) twice hourly and systemic antifungals. Four months after discharge, the patient returned with symptom recurrence.

**Conclusion:**

We report the case of a patient with a *Malbranchea* species causing a rare and recurrent fungal keratitis with corneal infiltrates, subsequently cured by medical management with salvaging of his vision. In patients with a suspected fungal keratitis, early treatment is crucial and should be combined with tight glycemic control for as long as 6 months after presentation to avoid recurrence.

## Highlights

• Fungal keratitis can lead to visual disability if not treated adequately.
• *Malbranchea* species are rare pathogens resistant to many antifungal agents.
• *Malbranchea* keratitis treatment is difficult and can result in poor outcomes.
• Treating fungal keratitis is an indolent process often requiring months.
• Standard protocols are necessary for managing fungal keratitis.

## 1 Introduction

Corneal blindness is the second leading cause of vision loss in developing countries. Unfortunately, unilateral corneal blindness is not always considered when estimating the burden of this disease. Primary prevention, early detection, standard first aid, and timely referral by primary care providers to ophthalmologists are key factors in reducing avoidable visual disabilities caused by corneal diseases [1]. The etiologies of unilateral corneal blindness include ocular trauma and infections. Standard protocols for management of bacterial and fungal keratitis have been well described [[2], [3], [4]]. Diabetes is a risk factor for development of fungal keratitis and can also hamper corneal healing, resulting in significant challenges during medical management [5]. In addition, abuse of contact lenses and corticosteroids have been shown to lead to increased rates of fungal keratitis and rare fungi affecting the cornea [6]. The management of this ailment requires collaboration between ophthalmologists, pathologists, and microbiologists.

*Malbranchea* species have been identified in the sinuses and lungs [7,8]. To the best of our knowledge, however, successful management of corneal infections caused by *Malbranchea* species have not been reported in the literature.

In this report, we share the case of a patient with a history of diabetes mellitus in whom this rare fungus caused an indolent keratitis that responded to prolonged treatment at our institution. This work has been reported in line with the SCARE 2018 criteria [9].

## 2 Case presentation

A 53-year-old male presented with a 14-day history of mild pain, redness, swelling, watery discharge, and a foreign-body sensation in his left eye (OS) following trauma with a wooden stick in a rural, semi-arid region. He arrived accompanied by his relatives in a personal vehicle. He had initially been treated by an ophthalmologist at a secondary-level eye care unit using topical natamycin 5% eye drops and moxifloxacin Q3 in the affected eye. However, despite 14 days of treatment, his symptoms had not abated, and he was referred to our university hospital for further management. The patient had an 18-year history of diabetes mellitus and was maintained on metformin 500 mg Q8 and insulin. Upon consult, his last HbA1c level was 9.3%.

**Fig. 1:**
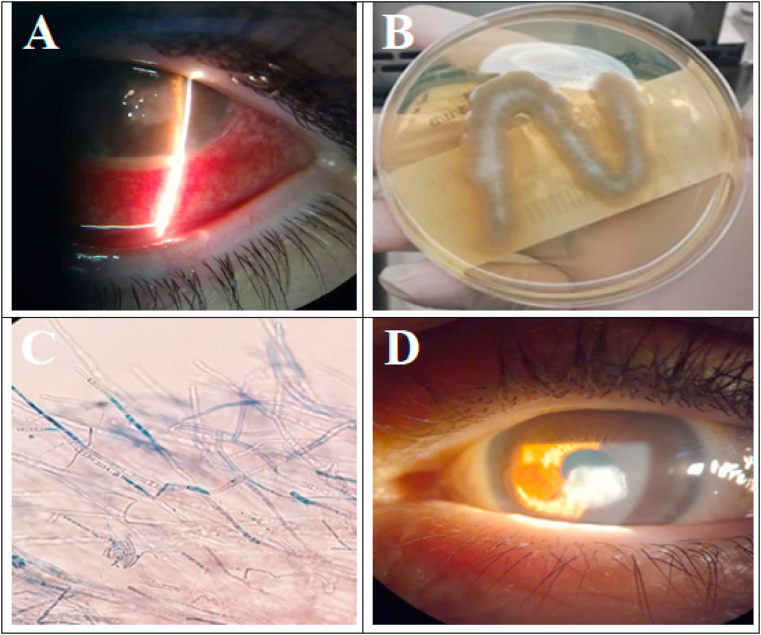
Organism profile and ocular status of a patient with a fungal keratitis caused by a *Malbranchea* species before and after 6 months of treatment. A – Slit-lamp examination showing congested conjunctiva with an overlying epithelial defect. B - Sabouraud agar showing growth of a cottony mold with a white-to-brown color. C - Microscopic examination showing thin septate hyaline hyphae without conidia or conidiophores. D - Paracentral corneal opacity 6 months after treatment of a fungal keratitis caused by a *Malbranchea* species.

On examination, the patient's best-corrected visual acuity (BCVA) was 20/40 in the right eye (OD) and 20/200 OS. The intraocular pressure (IOP) measured by a Tono-Pen (Medtronic, Minneapolis, MN, USA) was 15 mmHg OD and 14 mmHg OS. Slit-lamp examination (Topcon Healthcare, Oakland, NJ, USA), OS, revealed a diffusely congested conjunctiva and ring-shaped, grayish-white, central stromal infiltrates (1 × 2 mm) with an overlying epithelial defect and surrounding corneal edema. A corneal lesion with feathery margin and Descemet's membrane folding with a 1-mm hypopyon was also noted, OS (Fig. 1A). Anterior chamber reaction was present, with 2+ cells, flair, and a hazy view of the fundus. B-scan ultrasonography of the OS was anechoic. OD examination was unremarkable. Differential diagnoses included bacterial, viral, Acanthamoebal, or fungal keratitis; a provisional clinical diagnosis of fungal keratitis, OS was made. The patient was subsequently admitted for further management.

After applying topical anesthesia and ensuring aseptic precautions, the primary investigator (an attending ophthalmologist) took multiple scrapings from the corneal lesion's surface using a number 15 scalpel blade. The specimen was first smeared onto glass slides for Gram staining and a 10% KOH wet mount. Scrapings obtained were also inoculated directly onto solid media, namely blood agar, chocolate agar, Löwenstein-Jenson agar, and Sabouraud Dextrose Agar (SDA). These specimens were then transported to the microbiology laboratory for microscopy and fungal culture.

The patient was treated with topical natamycin 5% Q4 and atropine sulfate 1% eye drops Q8, alternative fortified antibiotics (ceftazidime and vancomycin) eye drops Q30 min, and intravenous paracetamol. He was monitored daily by a consultant ophthalmologist, and his diabetes was kept under strict control. Clinical improvement was noted after 2 days, with reduced conjunctival congestion and corneal infiltrates, as well as resolution of the hypopyon. This treatment regimen was based on our institution's guidelines for fungal keratitis treatment.

On the 5th day post-hospitalization, the microbiology report showed growth of a whitish-to-brownish cottony mold, appearing rapidly on SDA at 25 °C (Fig. 1B). Microscopically, thin, septate hyaline hyphae without conidia or conidiophores were noted. Arthroconidia alternating with empty cells were also present (Fig. 1C). All other cultures were negative. Primary identification suggested a *Malbranchea* species; final identification required confirmation by matrix-assisted laser desorption ionization-time of flight (MALDI-TOF) mass spectrometry. After diagnosis, the fortified antibiotics eye drops and natamycin were discontinued and topical voriconazole Q4 (1 mg/mL) and moxifloxacin 0.5% eye drops Q6 were started. Careful monitoring and strict diabetes control were maintained.

Fourteen days post-admission, despite treatment adherence and careful monitoring, the patient's clinical status progressively worsened. Repeat corneal scraping revealed a *Malbranchea* species on SDA culture. Itraconazole 200 mg was administered orally for 2 weeks, and topical amphotericin B (1 mg/mL) Q2 was also started.

On day 36 after hospitalization, repeat cultures were found to be negative. His symptoms had also resolved; the keratitis was believed to be controlled. He was thereafter discharged on topical amphotericin B (1 mg/mL) Q4 and two eye drops of prednisolone acetate 1% Q6 tapered over 6 and 4 weeks, respectively. He was also instructed about the importance of treatment compliance. The patient's eye and diabetes control were monitored weekly in the outpatient setting.

Four months after discharge, the patient returned with symptom recurrence despite treatment compliance. He had an HbA1c level of 8.5%. Again, he reported ocular pain, redness, and a foreign-body sensation; examination showed a 0.5mm hypopyon. This time, however, the corneal scrapings’ fungal culture was negative. The patient was treated with itraconazole 200 mg Q12 for 2 weeks and topical amphotericin B (1 mg/mL) Q4 for approximately 6 months. Over this period, the patient recovered without symptom recurrence.

At the patient's last follow-up, the HbA1c level was 7.5%, and BCVA was 20/30 with an IOP of 12 mmHg, OS. Slit-lamp examination revealed a corneal opacity without an epithelial defect or infiltration. His anterior chamber was clear, and the pupil was round and reactive to light. No ciliary congestion was identified with resolution of the keratitis (Fig. 1D). The patient was grateful he had successfully endured his long treatment course without residual visual deficits.

## 3 Discussion

In this report, we have presented the case of a patient diagnosed with a rare fungal infection causing long and recurrent episodes of keratitis. Due to his injury with a wooden stick and his history of uncontrolled diabetes, a fungal etiology was initially expected. Due to the recurrent nature of fungal infections, an urgent intervention was recommended in this patient. Delays in this patient's diagnostic work-up, including not collecting corneal ulcer specimens before initially starting conventional treatment at the secondary level eye care unit, were not ideal in this case.

In patients with diabetes, strict glycemic control, preferably under supervision, is crucial for controlling infections [10]. In addition, the importance of early microbiological testing cannot be overemphasized, especially in cases of recurrent keratitis. Though this patient responded well to supervised treatment during his first admission, it is possible that the transparent hyphae of this rare fungus remained under the healed epithelium before once again becoming active after his discharge. Based on our experience with this patient, we would therefore suggest that long-term antifungal treatment and strict glycemic control should continue even after the corneal ulcer is clinically healed.

During this patient's first hospitalization, his positive response to treatment could have resulted from both supervised glycemic control and use of natamycin for at least 15 days. At that time, no steroids were used to control inflammation. However, the initial positive response to this antifungal medication might have been eroded by mutations in the organism, leading to a need for parental therapies and the introduction of treatment with itraconazole and amphotericin B during recurrence.

The role of fungal infections in humans has not been widely studied. There seems to be a rising trend in the invasive nature of these saprophytic organisms, leading to increased numbers of deep tissue infections, especially in immunocompromised individuals [11,12].

## 4 Conclusion

In conclusion, we report a rare case of a patient in whom a *Malbranchea* species caused a recurrent fungal keratitis requiring vigorous antifungal treatments, both systemic and topical, which ended up healing without a scar. Based on our experience with this patient, we want to emphasize the importance of culturing corneal scrapings on fungal media, including SDA, as soon as possible in cases of suspected fungal keratitis. Moreover, early treatment of a suspected fungal keratitis and tight control of diabetes are crucial and should be continued for as long as 6 months after presentation to avoid recurrence. Larger case series or clinical trials are needed to identify a standard protocol for management of patients presenting with a red eye and visual impairment to secondary-level eye care providers, especially when a fungal keratitis is suspected. These rare types of organisms can only be detected if laboratory support is available and if samples are properly gathered. Moreover, conventional antifungal medications may not be effective in treating such rare fungi; therefore, treatment of fungal keratitis is an indolent process often requiring months.

## Ethical approval

The case report was approved by the local research and ethical committee (The IRB of King Abdulaziz City for Science and Technology).

IRB Number: 0383-24092

## Source of funding

The authors received no specific funding for this work.

## Author contribution

Ashjan Yousef Bamahfouz: Manuscript writing and review.

Abdulrahman Ali Alsaidi: Study concept, Data interpretation, Corresponding author

Ibrahim Jameel Alharbi: Data interpretation and manuscript writing.

Eman Abdulraheem Elsebaei: Microbiology report and review.

Ayat Mohammed Aldosari: Manuscript editing and review.

Ahmed Gamil Farahat: Concept design and literature review.

Renad Turki Alhazmi: literature review.

## Registration of Research Studies

Name of the registry:

https://www.researchregistry.com/

Unique Identifying number or registration ID:

researchregistry6159

Hyperlink to your specific registration (must be publicly accessible and will be checked):

https://www.researchregistry.com/register-now#home/registrationdetails/5f96a3acfa0fe7001509acfe/

## Guarantor

Ashjan Yousef Bamahfouz

Consultant Ophthalmology, Faculty of Medicine, Umm Al-Qura University, Makkah, Saudi Arabia.

E-Mail address: Ashjanmd@gmail.com

Phone/Mobile: +966547777059

## Consent information

Written informed consent was obtained from the patient for publication of this case report and the accompanying images. A copy of the written consent is available for review by the Editor-in-Chief of this journal on request.

## Provenance and peer review

Not commissioned, externally peer-reviewed.

## Declaration of competing interest

We know of no conflicts of interest associated with this publication, and there has been no significant financial support for this work that could have influenced its outcome.
